# Using Crowdsourced Trajectories for Automated OSM Data Entry Approach

**DOI:** 10.3390/s16091510

**Published:** 2016-09-15

**Authors:** Anahid Basiri, Pouria Amirian, Peter Mooney

**Affiliations:** 1 The Nottingham Geospatial Institute, The University of Nottingham, Nottingham NG7 2TU, UK; 2 Ordnance Survey GB, Southampton SO16 0AS, UK; Pouria.amirian@os.uk; 3 Department of Computer Science, Maynooth University, Maynooth W23 F2H6, Ireland; Peter.mooney@nuim.ie

**Keywords:** OpenStreetMap, completeness, spatial data quality, crowdsourcing, trajectory data mining

## Abstract

The concept of crowdsourcing is nowadays extensively used to refer to the collection of data and the generation of information by large groups of users/contributors. OpenStreetMap (OSM) is a very successful example of a crowd-sourced geospatial data project. Unfortunately, it is often the case that OSM contributor inputs (including geometry and attribute data inserts, deletions and updates) have been found to be inaccurate, incomplete, inconsistent or vague. This is due to several reasons which include: (1) many contributors with little experience or training in mapping and Geographic Information Systems (GIS); (2) not enough contributors familiar with the areas being mapped; (3) contributors having different interpretations of the attributes (tags) for specific features; (4) different levels of enthusiasm between mappers resulting in different number of tags for similar features and (5) the user-friendliness of the online user-interface where the underlying map can be viewed and edited. This paper suggests an automatic mechanism, which uses raw spatial data (trajectories of movements contributed by contributors to OSM) to minimise the uncertainty and impact of the above-mentioned issues. This approach takes the raw trajectory datasets as input and analyses them using data mining techniques. In addition, we extract some patterns and rules about the geometry and attributes of the recognised features for the purpose of insertion or editing of features in the OSM database. The underlying idea is that certain characteristics of user trajectories are directly linked to the geometry and the attributes of geographic features. Using these rules successfully results in the generation of new features with higher spatial quality which are subsequently automatically inserted into the OSM database.

## 1. Introduction

While traditional mapping is nearly exclusively coordinated and often also carried out by large organisations, crowdsourcing of geospatial data refers to generating a map database using Web 2.0 technologies. OpenStreetMap (OSM) is one of the most widely used and well-known examples of crowdsourced mapping and it has been widely used for a range of different applications such as navigation and humanitarian disaster response. Despite such broad usage of OSM, like most crowdsourced maps, the completeness, reliability and the accuracy of OSM data have been questioned [1,2,3,4,5,6,7,8]. 

The issues related to the quality of OSM data are often linked to the OSM project having many contributors who have had little or very limited training with collecting and managing geospatial data [9,10,11]. Additionally, there are many areas and features in the OSM database which have been mapped by visitors, tourists and people who do not live in or have local knowledge of the area(s) they edit. These “non-resident” mappers often have a low level of familiarity with these mapping areas. This has been shown to contribute to lowering the semantic and temporal quality of the data [12]. It can also result in inserting geometrical objects without sufficient descriptive attributions [13]. 

Contributors with different nationality, language proficiency and preferences, and cultural backgrounds can have different interpretations and understanding of the meanings of the attributes and tags used within OSM. Such differences between contributors can mean the OSM database contains heavily edited features or features containing their geometry with only a minimum number of attributes or tags to describe them [14,15,16]. These issues can also be introduced where the contributors have different levels of enthusiasm for the mapping tasks at hand. Some “less-active” mappers can quickly disengage from the OSM project after only completing a small number of contributions. This can contribute to the large number of features in the OSM database which have little or no attributes or tags attached to them [13,17,18]. In this regard, the online user-interface for viewing the OSM map and editing the OSM database can often lead to confusing many contributors even more [19].

All of these contributor-related sources of uncertainty in the OSM database could be improved if the direct interactions between the contributors and the OSM database were minimised. Direct data entry and editing of features in the OSM database require mapping contributors to have a relatively good understanding of the features and their descriptive attributions [20]. Poor understanding can cause problems for the quality and completeness of OSM data. These problems can be mitigated if the direct involvement of the mappers in the data manipulation process could be minimised.

**Figure 1:**
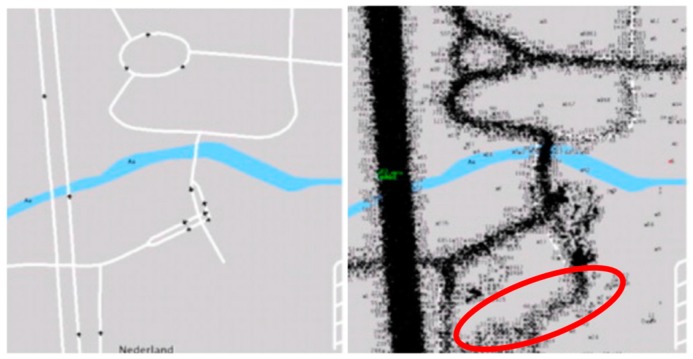
Detection of a missing road on the map using the trajectories of users.

This paper presents a new approach whereby during data entry, the new geographic data can be added and be used to extract the common rules and patterns which can be linked to geometric and attribute data of features. This mechanism can identify and store the features with commonly agreed attributes and tags attached to them. This approach is based on data mining techniques, which use large amounts of raw data (i.e., trajectories of movements) and the rules and patterns corresponding to many common scenarios. This mechanism can then lower the likelihood of inserting features with low positional, temporal and thematic accuracy and completeness of coverage. The areas in OSM, which have been mapped by the larger number of active contributors, usually have overall better data quality [21]. The use of trajectory mining for identification of incorrectly tagged features in OSM datasets has been proposed and used by [22]. Their methodology extracts wrongly tagged OSM features based on the rules and patterns extracted from trajectories. However, their method can only work if the underlying feature is already inserted by a mapper with some descriptive attributes attached to it. In another words, their methodology can only detect bugs and errors in the OSM attribute data. The methodology cannot insert geometry or add a new tag to the OSM database. The method proposed in this paper can automatically recognise potential features and their attributes based on the patterns and rules extracted from trajectories of movement. As a result, new geometrical and attribute data can be inserted into to OSM database. This automation of high-quality data entry can provide answers to the completeness and the coverage issues of OSM data in many areas which currently have poor quality OSM data. Figure 1 shows an area where the vertices of the trajectories (right side of the figure) show the existence of some features while the underlying map (left side of the figure) shows no road feature.

In addition to the improvement in the overall completeness and coverage of OSM data [8] the data entry process can also be improved. The capture of this input raw data is much less demanding than actual map data insertion. The input of the raw data requires almost no experience and expertise on the part of the contributor. In order to have an even higher level of accuracy and reliability the result of the proposed methodology can be stored for an expert-validation phase [15] or for comparing with other sources of data (e.g., Google Maps and the Ordnance Survey GB). Alternatively, it can simply be added to the OSM database and marked for “second opinions” for further quality assessment by other OSM contributors.

The idea presented in this paper is to analyse contributors’ travel behaviours to derive rules which can then be used for feature identification and attribute inference. This paper focuses on inserting and editing (including the update and deletion of) features and their tags based on spatial knowledge automatically extracted from the anonymous tracking data contributed by OSM users. The quality validation of already existing features in OSM data is not discussed in this paper. Most of the OSM map editing interfaces and map editing software tools provide simple mechanisms for OSM contributors to upload GPS traces or trajectory datasets.

It is possible to learn rules and recognise patterns from these trajectories and use them for automated feature identification and attribute inference. Such patterns and rules may highlight some clusters and bundles of trajectories, which may represent some real-world features such as roads [23], buildings, and parking spaces [24,25], and/or indicate some of their attributes [26,27,28]. For example, it is possible to recognise the type of a road, based on the statistical analysis (e.g., average/minimum/maximum speed) of the clusters of the trajectories’ segments [29]. Another example is to estimate the capacity of parking, based on the number of concurrent trajectories ending or having significant stay points at a parking space [30]. Inference about the type of the buildings, such as restaurants and shopping centres, is based on observing the spatio-temporal characteristics of the visits (such as time of the visit for the customers) [25,31]. In order to recognise such patterns and rules in these data an inference engine was developed as an ArcGIS add-in, to store, visualise, and analyse trajectories using spatio-temporal data mining techniques. 

The next section explains how an automated data entry process can improve the quality and completeness of data within the OSM database. In the third section, the details of the proposed methodology and workflow are discussed, along with the size and quality requirements for the input data. Section 4 shows the implementations and the achieved results. 

## 2. Why Is an Automated Data Entry Process Required?

It is reasonable to assume that the completeness and the quality of OSM data can be improved if the errors and bugs inserted by non-experts and untrained contributors are minimised. This can be achieved by an automated data entry process, based on raw data, which does not need any interpretation by these users. This section explains why an automated data entry procedure is required to improve the completeness and the quality of OSM data.

Volunteered Geographical Information (VGI) is a source of geographical information in which there is no definite traditional boundary between the authoritative map producers and the public map consumers [32]. The terms VGI and crowdsourced geographical data are interchangeably used in the literature [33]. One of the most successful projects based on crowdsourced VGI is OpenStreetMap (OSM) which started in 2004 as a project and has increasingly attracted contributors over this time. Users or contributors to OSM may map the world using a variety of techniques such as GPS traces or their local knowledge assisted with some aerial imagery [34]. Moreover, with the OSM data the unrestricted application of key-value pairs (tags) for tagging and annotating features provides an excellent means of customised annotations suitable for many thematic applications. A complete review of recent developments in OSM is available in [35]. At the time of writing, there are over 2.6 million registered users, more than 3.6 billion objects and 1.3 billion tags in the OSM database [36]. 

**Figure 2:**
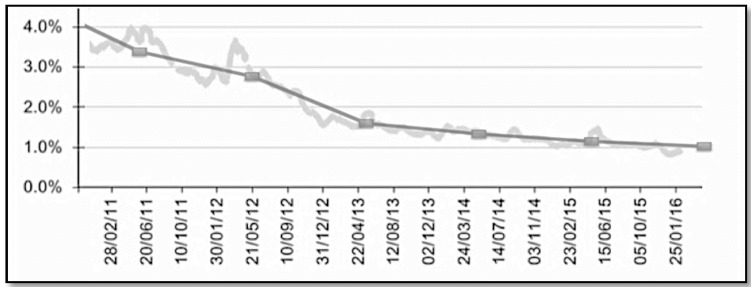
Percentage of the active contributors relative to total number of accounts.

As it is shown in Figure 2, the majority of the newly registered members in OSM are not always “active contributors”. At the time of writing, only 0.85% of all registered OSM members can be considered as active contributors. During this time more and more non-active members have joined OSM while the growth rate of new active members or contributors is decreasing as shown in Figure 2. This is not necessarily a negative factor. This can potentially be interpreted as a success factor for the OSM project demonstrating that OSM can attract ordinary people, as their main contributor base, whose main activity or skill is not mapping but are mapping local features in OSM as a hobby. Attracting a mainly non geospatially skilled contributor based means that user interface simplifications are required, data quality assurance processes must have easy to follow instructions, etc. In terms of where new contributors come from, there is a lack of new contributors from countries with poor OSM data coverage. Developed countries still see the most of edits being carried. Excluding a few exceptions, where there have been global calls for humanitarian actions for mapping such as the recent Ebola outbreak in West Africa [37], there is a lack of OSM contributors in developing countries. Whereas external contributors can help with up-to-date new entries, these poorly mapped areas continue to suffer from insufficient numbers of active and local contributors who have an interest in mapping these areas from scratch.

The majority of the contributors are citizens with no significant expertise in mapping, surveying, and geospatial data. When this is combined with a lack of active local contributors we suggest that OSM may have to consider some of the following features, including:

- Spatial quality assurance measures: due to the free and open nature of OSM there is a need to check the quality of OSM data on an ongoing basis. As reviewed by [22] there are three main categories of OSM quality control: (a) comparing OSM data against authoritative spatial data; (b) user and rule-based checking; and (c) crowdsourced rule and pattern extraction for rule-based checking.
- Easily interactive and user-friendly mapping interfaces: When the majority of OSM contributors are not part of the ‘active mappers’ group, levels of enthusiasm can vary dramatically and some may get bored and unengaged faster. In this regard, the online user-interface, where the underlying map can be viewed and edited, can both help or confuse these contributors. In addition to this, the introduction of more easily interactive and less complicated approaches for data entry and editing can help users to spend more time on mapping which can potentially result in better quality and completeness of OSM data.
- Easy to follow procedures for data entry: mapping procedures which require a minimum of experience, skills, human-interaction and overall dedicated time, can help non-experts to contribute more frequently.

An additional solution proposed in this paper is based on minimising the direct interaction of contributors with the OSM database. This solution is based on the contribution of members’ raw geographical data, such as trajectories/traces of their travel. An automated workflow could identify geometrical features and/or attributes from contributors’ raw data and subsequently automate the data manipulation process. This minimises the direct involvement of inexperienced contributors in the manipulation of features within the OSM database and associated processes. We expect that using this solution will go a long way towards reducing the number of issues related to low-quality data entered into the OSM database. If this automated procedure could obtain the raw data from contributors and then pre-process these data to filter low-quality data there is the potential to make the attribute data more consistent. It could also then be re-applied to many poorly mapped areas where there are currently not enough active mappers. 

## 3. Methodology

In this section, we outline the methodology for an automated mechanism which generates OSM nodes, ways and/or tags, i.e., features with both geometry and attributes, directly from the trajectories of movements provided by contributors. This mechanism is based on mining and analysing a large amount of the Global Navigation Satellite System (GNSS) tracks, contributed by OSM members. The trajectories are analysed and the rules and patterns, which can be an indication for the geometrical and attribute aspects of real-world objects, are recognised.

**Figure 3:**
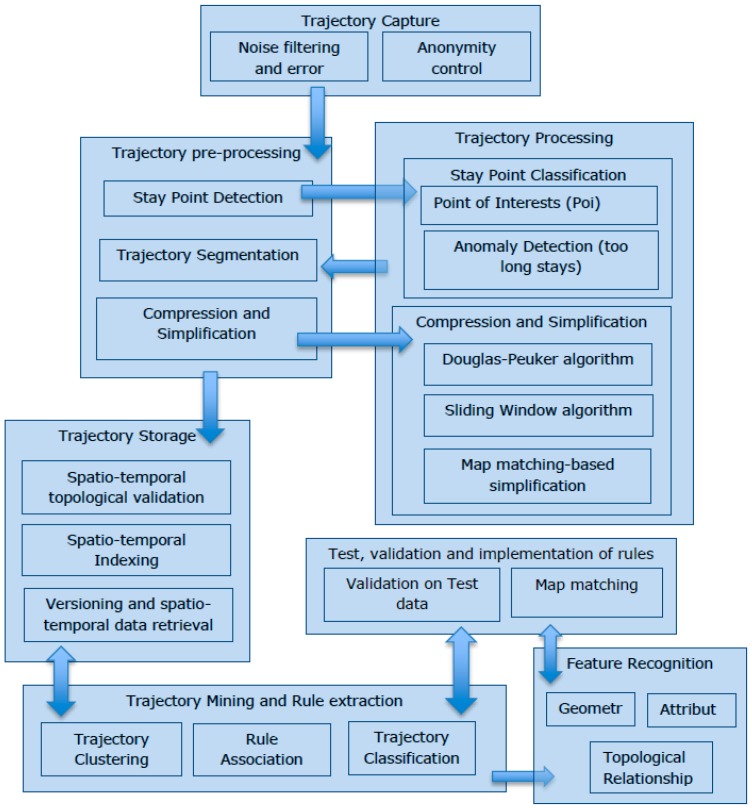
High-level of abstraction of the feature recognition and attribute allocation process based on trajectory mining.

At the time of writing, there are more than five billion Global Positioning System (GPS) points stored in the OSM database. Such large volumes of uploaded GNSS data shows the ease and convenience of data capture and can potentially provide our proposed procedure with a rich data source for training and testing. In addition, the automation of the data entry process could help to improve the perceived reliability of OSM data. The belief that OSM is created by amateurs is often seen as a barrier to trust and acceptance of this free data source within the traditional GIS community. The methodology described here can also be applied to features already existing in the OSM data. This allows the extraction of additional tags and can improve the thematic completeness of the OSM data overall. At present, the number of tags assigned to nodes or ways in OSM is rather small. On average, each node and way can have 3.22 and 2.75 tags for annotation purposes, respectively. The proposed mechanism can infer additional attributes and increase the number of tags describing the OSM objects. This workflow process is illustrated in Figure 3.

As shown in the proposed workflow, trajectories of movement are firstly anonymised with errors and noisy data points removed. Then within the trajectory pre-processing step, the trajectories are broken down into segments and compressed. Some trajectory segments can have more semantically enriched data. For example these segments can include *stay points* where users have stayed for a while other segments have lots of data points and can be further simplified. Similarly, the output of the pre-processing step can also undergo further processing. The simplified and compressed segments along with the stay points are stored in a database. Then using spatio-temporal data mining techniques, some clusters of trajectories can be identified that share some common behaviours, e.g., similarity in the speed and the direction of segments. Such behaviours, extracted as rules and patterns, can indicate some of the real-world features (both their geometry and attributes). The extracted rules and patterns, from trained data, are tested over control data. If the recognised rules and patterns pass both the control and validation step then these rules can be applied for data entry. In the following subsections, each step and the applied algorithms and methods of our proposed methodology are explained in detail.

### 3.1. Input Data Preparation and Storage

The inputs to the workflow are the trajectories of movements contributed by registered OSM members. Like the OSM data entry process, these contributors can upload the GNSS (such as GPS or GLObal Navigation Satellite System (GLONASS)) traces to the available repository. The available GNSS traces from the OSM database can also be used for this approach. One of the easiest ways of OSM data entry and editing is to upload GNSS traces. The GNSS traces can be recorded by mobile devices and In-Vehicle Sat-Nav systems or almost any other device equipped with GNSS receivers. The GNSS points can be stored at a different time interval (e.g., every second) or distance (e.g., every meter (trace log)). Many GNSS-enabled devices can directly convert data into the GPX format. Contributors, however, can upload their traces in other formats. In the pre-processing phase the format conversion is performed so that contributors do not need to convert their raw data into GPX.

Although uploading the GNSS traces is one of most common ways of uploading data for our proposed approach a range of different positioning and tracking technologies and methods are accepted including: GNSS, Wireless Local Area Network (WLAN), cameras, mobile networks, Bluetooth networks, tactile floors and Ultra Wide Band (UWB) [38], or even drawing a line to show the trajectory of movement (without mentioning the important locations visited).

The first step in data preparation, as it is shown in Figure 3, is anonymity control and noise filtering. In order to keep the anonymity of the trajectories the identity of the contributors must not be linked to the data and the pseudo-name selected by the contributors. In addition to this, a trusted third party software package is used on the tracking data. While there are many available anonymisers [39], this paper uses the K-anonymity program [40].

Anonymised trajectories can have some points that are not perfectly accurate or in some cases even valid. Such errors and noises should be filtered in advance to minimise the invalid outputs at the end of the data mining process. It is very important to remember that this phase is being carried out to filter the errors and noises and not to exclude the abnormalities and anomalies that might be helpful for some applications and scenarios. Noises and errors in the data exist due to several reasons such as poor and multipath positioning signals. In order to detect and exclude noises and errors, there are methods available, such as Kalman and Particle filtering and mean (or median) filters, described and reviewed by [41]. This paper uses a heuristic-based method: other filters replace the noise/error in the trajectory with an estimated value and this may have a significant impact on the output of trajectory mining (i.e., the recognised patterns and rules). Our methodology calculates the distance and the travel time between each consecutive pair of points in the trajectory. Subsequently, the travel speed for each segment can easily be calculated. It is then possible to ascertain the segments whose travel speeds are larger than a threshold (e.g., 360 km/h). 

If the travel mode for each trajectory is also identifiable (identified by the contributor or based on statistical methods) we try to find the consecutive segments with almost the same average speed. These segments are then classifiable into three classes of travel: pedestrian, car/bus/ train and bike. It is then is possible to have different thresholds depending on the travel mode (e.g., a pedestrian cannot walk faster than 20 km/h.) Our methodology only uses these travel modes if they are specified by the contributors. Otherwise, the methodology simply ignores the possibility of error/noise detection using the statistical methods. This is due to the existence of the transitional segments (e.g., from pedestrian to car and then again to pedestrian mode) or the anomalies (which are not due to errors or noises). If anomalies or transitional segments are removed then some valuable information can be lost. Such segments need to be retained carefully for the next steps of the trajectory data mining process as they can potentially contain valuable information. 

In addition to the above-mentioned heuristic-based method our methodology uses the ensemble methods [42], in addition to various rules of thumb and predictive rules, to identify anomalies. A large number of points were labelled to produce a large training set. If a large number of classifiers with an accuracy measurement slightly better than a random guess is combined together then the accuracy of the ensemble is superior to most known classification algorithms. The idea of ensemble learning is to build a prediction model by combining the strengths of a collection of simpler base models [43,44].

Most ensemble models in off-the-shelf software packages use binary decision trees as the weak learners and produce an ensemble of hundreds or thousands of binary decision trees. However, this work uses a novel approach for combining other types of classification methods, in addition to binary decision trees. In MajorityVoteEnsembleClassifier it is possible to use powerful classification algorithms like logistic regression and support vector machines and subsequently improve their accuracy by using a combination of these models.

**Figure 4:**
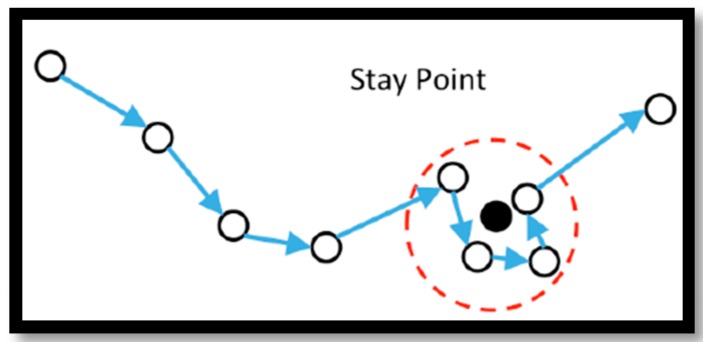
Stay point where the users have stayed for a while at (almost) the same location.

The second step is trajectory pre-processing. The pre-processing step is to make trajectories (1) easier to store (using segmentation, compression and simplification techniques) and (2) potentially semantically meaningful (by identifying the stay points). The pre-processing step is actually based on the fact that all the points in a trajectory are not equally important and meaningful [45]. The pre-processing step makes the trajectories ready to be stored and retrieved in a more efficient way. In addition to efficiency, trajectory segmentation and stay point detection make the trajectories and some of the points semantically meaningful and easier to interpret. Stay points refer to the locations where users/contributors have stayed for a while. The stay points can simply be identified if the location of the user remains fixed over a specific period of time. However, due to the inaccuracy of some positioning technologies, even if the user is not moving the position could be different, see Figure 4. 

In order to detect stay points, there are several algorithms and methods; In [46] an approach that checks the travel speed for each segment was proposed and if it is smaller than a given threshold then the average of these two points are replaced/stored as the “stay point”. The distance and temporal interval threshold can be set separately rather than the speed of each segment; for example, if the distance between a point and its successor is larger than the set threshold and the time span is larger than a given value, as proposed in [47]. Reference [48] proposed the use of a density-clustering algorithm to identify the stay points, which is used in this paper to identify the speed clustering algorithms and thus the stay points. This can be viewed as the combination of segments’ speed threshold and the density-clustering algorithms.

**Figure 5:**
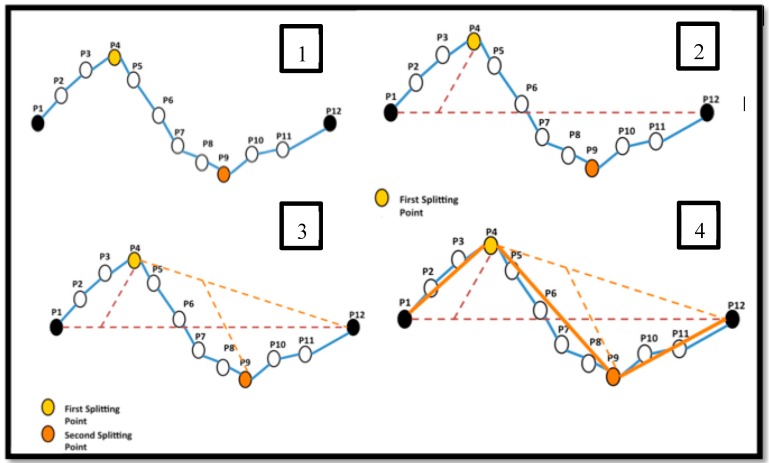
The extended Douglas–Peucker algorithm taking distance and time for simplification and segmentation of trajectories.

In order to better manage the trajectories and also exclude small changes in movement, which are mainly due to inaccuracy and imprecision of the positioning technologies, the trajectories are simplified. For simplification and segmentation of the trajectories, we extend the traditional Douglas–Peucker algorithm [49]. This algorithm takes a combination of distance and time, instead of only distance, to identify the splitting points. This means the splitting points are identified where the speed of the movement over segments, i.e., a combination of distance and time rather than only distance, becomes greater than the threshold. This simplification reduces the overall volume of the data and also improves the performance of the clustering of these trajectories. As it is shown in Figure 5, the distance of each point from the baseline, connecting the start and the end of each trajectory, multiplied by the inverse of the corresponding time interval, are calculated. If this value is greater than a threshold then this point is considered as a splitting point. 

Pre-processing the input data enables its storage in a spatio-temporal database [50] and subsequently retrieved for further analysis and data mining [46]. Although data mining techniques are well-developed, in order to have a better pattern recognition process and consider all aspects of trajectory data, it is highly recommended to apply spatio-temporal data mining techniques, to consider the spatio-temporal relationships through this process.

### 3.2. Pattern Recognition and Rule Extraction 

In order to extract rules and patterns, spatio-temporal data mining techniques are used. As discussed by [51], the forms that spatio-temporal rules may take are extensions of their static counterparts. However, at the same time they are uniquely different from them. Five main types can be identified:

- Spatio-Temporal Associations. These are similar in concept to their static counterparts as described by [52]. Association Rules are in the form X → Y (c%, s%) where the occurrence of X is accompanied by the occurrence of Y in c% of cases (while X and Y occur together in a transaction in s% of cases).
- Spatio-Temporal Generalisation. This is a process whereby concept hierarchies are used to aggregate data, thus allowing stronger rules to be developed at the expense of specificity. Two types are discussed in the literature: spatial-data-dominant generalisation proceeds by first ascending spatial hierarchies and then generalising attribute data by region. Non-spatial-data-dominant generalisation proceeds by first ascending the spatial attribute hierarchies. For each of these types, different rules may result.
- Spatio-Temporal Clustering. While the complexity is far higher than its static non-spatial counterpart the ideas behind spatio-temporal clustering are similar. In this case, either characteristic features of objects in a spatio-temporal region or the spatio-temporal characteristics of a set of objects are sought.
- Evolution Rules. This form of rule has an explicit temporal and spatial context and describes the manner in which spatial entities change over time. Due to the exponential number of rules that can be generated, it requires the explicit adoption of sets of predicates that are usable and understandable. Example predicates include Follows, Coincides, Parallels and Mutates [53,54].
- Meta-Rules. These are created when rule sets rather than datasets are inspected for trends and coincidental behaviour. They describe observations discovered amongst sets of rules. For example, the support for the suggestion that X is increasing. This form of rule is particularly useful for temporal and spatio-temporal knowledge discovery.

In order for these to give valid results, the input datasets need to satisfy some preconditions. If the input dataset is small (in terms of the number of trajectory samples, the extent of the trajectory areas and the density/frequency of trajectories (with respect to space and time)), then outputs (the rules and patterns) may be only valid for a specific situation, area, and time interval. The robustness of output knowledge is strongly correlated with input sample size. The input samples need to be large, otherwise, the training and control/test processes may not find all patterns hidden in the data. The input samples should also be dense and frequent enough to give complete spatial and temporal coverage, (different days, times, weekends, seasons, and so on). Finally, the samples should also cover all (at least most) travel behaviours, travel modes and building types to give a more reliable overall tagging inference.

The above-mentioned preconditions might qualify the data to be analysed by spatio-temporal data mining techniques to identify the patterns, clusters, and rules which can highlight the features the users travelled through, to, from, visited or were at. 

One of the most important analyses, which is the basis of some rule associations and pattern recognition, is trajectory clustering. A general clustering approach represents a trajectory with a feature vector. This denotes the similarity between trajectories by the distance between their feature vectors. The methodology in this paper proposes a clustering method that can be applied to the trajectories in free spaces (i.e., without a road network or map constraint). This is due to the obvious fact that the base map/features are not available to do map-matching. This paper uses the Hausdorff Distance metric to identify similarity with an adopted Micro- and Macro-clustering framework. In the CTHD, the similarity between trajectories is measured by their respective Hausdorff distances and they are clustered by the DBSCAN clustering algorithm [55].

Such clusters are being used to identify patterns of movement. For pattern recognition and rule associations we need to identify the group of people that move together for a certain time interval. These groups can be considered as: (1) a flock (i.e., a group of objects that travel together within a geographic disk of some user-specified size for at least k consecutive time stamps); (2) a convoy (a group of objects that are density connected during k consecutive time stamps); (3) a swarm (a cluster of objects lasting for at least k (possibly non-consecutive) time stamps); (4) a travelling companion, which uses a data structure (called travelling buddy) to continuously find convoy/swarm-like patterns [56]. These patterns can help to understand some of the feature attributes, such as the type of the building (e.g., residential, office, restaurant), traffic lights, bus lanes and the detection of celebrations and parades.

A cluster of trajectories, with an average speed of, for example, 63 mph and an average length of 2 miles, with no crossing cluster (which could show a junction), can highlight a segment of a motorway. In this example, the type of the road is inferred from the speed of the movement and the length of the trajectories in a cluster. However, at a relatively lower speed, there might be a higher level of uncertainty in such attribute classification, due to a lack of a clear distinction in the classification of the feature to carriageways, motorways and A and B type roads (in the UK system). In such situations, some additional data, such as the width of the road that can be estimated using the maximum distance of trajectories in a road must also be deduced. Another example is the identification of residential buildings. These can be identified if a cluster of trajectories gathers at one (relatively small) area, in particular in the evening. This pattern remains unchanged during the night and the number of trajectories in the cluster is not high, i.e., the number of residents.

For the objects identified as roads, the tags regarding the direction (either one-way or two-way road) can be inferred; if travel is in both directions, based on the headings of the GNSS points, the road can be classed as one-way and two-way. However, this error and bug detection approach uses a conservative strategy; the robustness of the rules and the reliability of the results depend highly on the application requirements, the spatial, temporal coverage and the size of training and test datasets. 

Finding objects and attributes using inferred rules and learned patterns from crowd-sourced data is likely to have valid real-world results since the rules are based on actual behaviours. This approach extracts rules and patterns from trajectories which are more adaptable to the domain since several pieces of information can be potentially extracted from the trajectories. Contrast this with a user-entry approach where contributors directly insert and edit OSM data, including geometry and attributes. This approach is based on their own knowledge and understanding of OSM, thematic domain, local tagging approaches, etc. This knowledge might not be correct or even up-to-date. This can cause a number of contradictory edits from different users who have different understandings of the meaning of tags and attributes and different knowledge of the spatial accuracy of features, and so on. Using automatically captured trajectories means it is possible to extract such knowledge and take action (edit, delete, or insert a feature) where large enough samples of trajectories support the inferred knowledge and pattern. The description of the steps of the proposed process and the implementation are explained in the next section.

## 4. Implementation and Results

**Figure 6:**
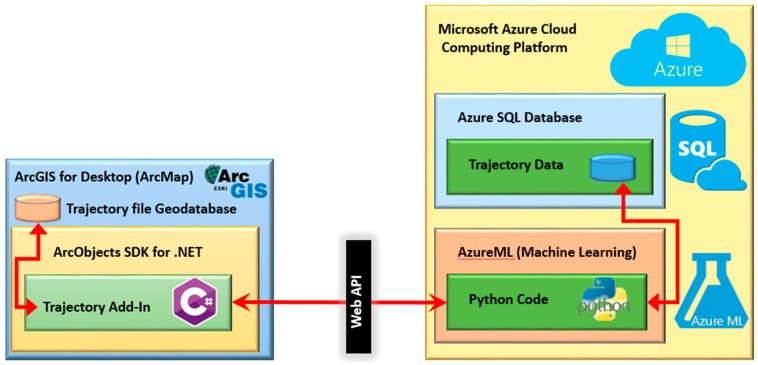
Trajectory analyser dockable window (developed ArcGIS add-in).

The workflow of our proposed methodology is implemented as an ArcGIS add-in. As illustrated in Figure 6, data mining and machine learning functionalities are hosted within Microsoft Azure cloud computing platform. Geospatial functionalities (such as visualisation, generalisation) are implemented as an ArcGIS add-in. Communications between the cloud-hosted machine learning functionality and the ArcGIS are performed over the Web.

The workflow takes the trajectory data as the input, and then it stores the “potential objects” in a feature dataset as an output. Contributors provide their movement trajectories to a centralised repository and an inference system finds patterns in the data and derives inherent rules which can be used to identify the geometry and attribute of features they have visited or been to. Such patterns and rules highlight features, which may need to be examined (for further quality assurance) and stored as “potential objects”. If these potential objects are matched against already existing OSM objects then additional attributes can be inferred and added. 

**Figure 7:**
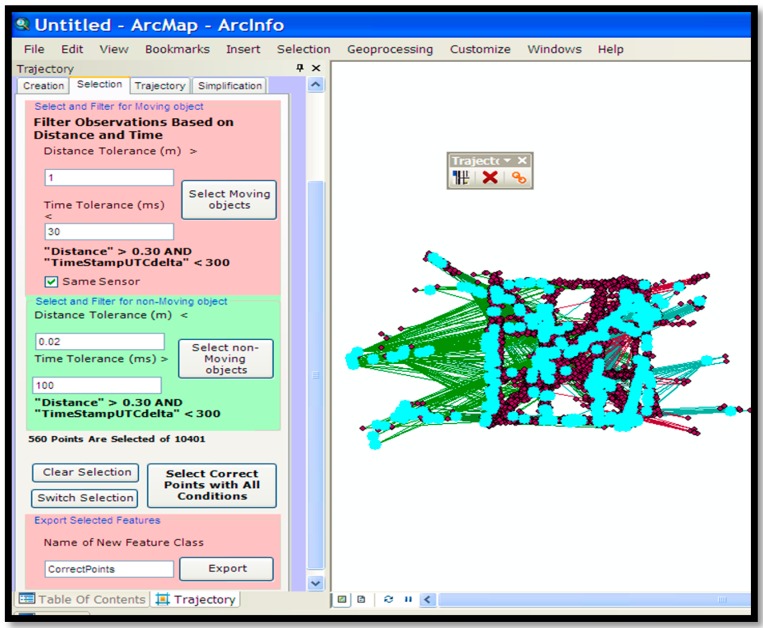
Noise/error filtering and stay point detection tab.

As previously mentioned, an ArcGIS add-in has been developed (see Figure 7) to visualise, process and analyse the input trajectory data. As Figure 7 illustrates, a trajectory analyser in a dockable window is available to ArcMap. The first tab creates a feature class by reading the input XML file containing all of the points of all traces.

Although the trajectories are already (pseudo) anonymised and there is only a user ID attached to each object, due to privacy and data protection issues the link between the trajectory data and the OSM ID/reference to the contributor is removed and the data is stored without any reference to the OSM member. This paper uses the K-anonymity program available by a trusted third party software package.

In order to remove noises and errors from input data a heuristic-based method is used. This method identifies the abnormal segments whose length and/or the travel time is too high, i.e., greater than a given threshold. This threshold is set to 360 km/h which is greater than the speed of the ordinary vehicles on the roads and well above accepted speed limits on roads and motorways. However this value can be easily changed as it is shown in Figure 5. The noises and errors within the consecutive couples of points (start and end points of the segments) are identified. 

**Figure 8:**
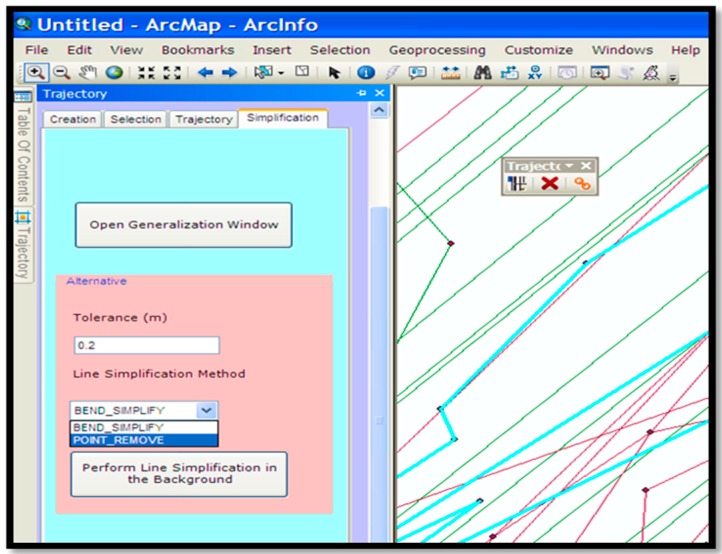
Simplification tab based on the extended Douglas–Peucker algorithm.

The ArcGIS add-in can add two columns to the FeatureClass, which contains the distance between each adjacent pair of points (the length of each segment) and the speed of the movement of the user inferred from the trajectory’s statistical information or from passing that segment. The travel speed is compared against the threshold. This threshold is set based on the statistical information (average speed) of the trajectories having the same travel mode. If the travel mode is not specified by the contributor then the speed is set to 360 km/h. However, this value is an expert-defined value and can be easily changed as shown in Figure 8. The travel speed is stored as attribute data for each segment as this information can be used at a later stage for rule association and pattern recognition steps. This paper uses the ensemble methods, in addition to various rules of thumb and predictive rules, to identify anomalies. This paper uses a novel approach for combining other types of classification methods as well as binary decision trees. In MajorityVoteEnsembleClassifier, it is possible to use powerful classification algorithms like logistic regression and support vector machines and subsequently improve their accuracy by using a combination of these models. The developed Python code implements some machine learning algorithms (such as MajorityVoteEnsembleClassifier, is explained later) by extending the scikit-learn Python package. The scikit-learn package is the most widely used machine learning package in the Python programming language. By inheriting from the BaseEstimator class in the scikit-learn package the implemented algorithms inherit all methods similar to the other models within scikit-learn. 

The Python code is then hosted in the AzureML service of the Microsoft Azure cloud computing platform. Microsoft’s Azure Machine Learning (AzureML) dramatically simplifies machine learning model deployment by enabling data scientists to deploy their final models as web services that can be invoked by any application on any platform, including desktop, smartphone, mobile and wearable devices [57]. Hosting the Python code for this research project in AzureML allows us to use the same model from various platforms without worrying about scalability issues. Data for training the models are stored in a SQL Azure database which is the trajectory database.

The C# programming language was used for implementing the ArcGIS add-in. ArcGIS Desktop add-ins are the preferred way to customise and extend ArcGIS for Desktop applications. With add-ins all the functionality of ArcGIS for Desktop applications is available through ArcObjects which is set of components that constitute the ArcGIS platform [58]. The Trajectory add-in is implemented as a Dockable window in ArcMap. The first tab creates a feature class in a local file geodatabase by reading the input XML file of the trajectory points.

In summary, this algorithm can be used as an anomaly detection algorithm when the training data are composed of points with two labels (valid and invalid-anomaly). It is also possible to provide weights for input training datasets in order to learn from the mistakes of previous classifiers in different iterations.

In addition to the classification of the trajectories’ segments, the MajorityVoteEnsembleClassifier also helps to exclude redundant data. For example it allows us to find points where the user has been stationary (speed close to zero) and replaces them with a single point with a description of the time interval during which the user’s speed was zero.

By calculating the correlation between trajectory data it is possible to discover other modes of classification. Spatio-temporal clustering helps to identify such classes and discover underlying rules and patterns through identifying parameters which are highly correlated and then extracting rules. Evolution rules are applied at this stage through functions on the selection tab to discover spatial and temporal rules.

### Trajectory Pre-Processing

The next step is data pre-processing. As explained previously the identification of the stay points and split vertices must be done first. This paper computes the travel speed for each segment and if it is smaller than a given threshold then the average of these two points are replaced/stored as the “stay point”. The distance and temporal interval threshold can be set separately instead of the speed of each segment. These are set separately if the distance between a point and its successor is larger than the set threshold and also the time span is larger than a given threshold value. This can be viewed as the combination of segments’ speed threshold and the density-clustering algorithms. For simplification purposes, which is essential for efficient data management, the extended Douglas–Peucker algorithm, explained in Section 3 is used. The implementation of this algorithm in the developed ArcGIS add-in is shown in Figure 8.

For the purpose of trajectory clustering, which is the basis of the rule extraction and feature recognition parts, this paper uses the Hausdorff Distance metric to identify similarity with an adopted Micro- and Macro-clustering framework. This is mainly due to having the sample free trajectories. The trajectories are not bound to the road networks nor do they come with different shapes and the number of vertices (even for the same area). Given this situation, we had to use the clustering methods that can be applied to the trajectories in free spaces (i.e., without a road network or map constraint). Map-matching based algorithms could not be used as the methodology in this paper tries to identify new features that are not already stored.

This paper uses the Hausdorff Distance metric to identify similarity with an adopted Micro- and Macro-clustering framework. This method distinguishes the trajectories in different directions, micro clusters and macro clusters [55].

Such clusters are used to identify patterns of movement. For pattern recognition and rule associations, we need to identify the group of people that move together for a certain time interval, such as a flock, a convoy, a swarm, and a travelling companion. These patterns can help to understand some of the feature attributes, such as the type of the building (e.g., residential, office, restaurant), traffic lights, bus lanes and the detection of celebrations and parades.

In order to extract patterns and rules of movements, all input trajectory data are randomly divided into two feature classes. The first one which is called training data is used for pattern recognition and rule learning purposes. Another set of input data, which is called control data, is used to control how the learned rules and recognised patterns fit into this set of data. After analysing and finding patterns in the training data, the inference system will apply the extracted patterns on the control data to see how similar input control data and estimated results are fitting. If they are very similar, it is possible to infer that a pattern was discovered and any new data can be analysed using that pattern. 

The clusters and classes which can be generated using above-mentioned techniques are used to generate rule sets. The final step of knowledge discovery from data is to verify that the patterns produced by the data mining algorithms occur in the wider dataset. Not all patterns found by the data mining algorithms are necessarily valid. It is common for data mining algorithms to find patterns in the training set which are not present in the general dataset. 

If the learned patterns do not meet the desired standards then it is necessary to re-evaluate and change the pre-processing and data mining steps. If the learned patterns actually do meet the desired standards then the final step is to interpret the outputted learned patterns and turn these into actionable knowledge.

In this paper, since prior knowledge about the input data was included (such as a reference map or additional spatial data), it was decided to evaluate the correctness and logic of output patterns and rules using standards, “common sense” rules-of-thumb and expert comments as well as control data test. However for the purposes of verification there is a need to compare the results of this approach with other approaches to evaluate them. One of the early inferred rules and patterns is about identifying the travel mode using only speed and behaviour of movement. Based on the speed of the movement it is possible to classify data into the four categories of pedestrian, bike, wheelchair and vehicle. Using patterns of movement it is possible to find some rules which distinguish between public transportation and cars. For example, public transportation stops regularly at very specific points with very low correlation to time as whenever a vehicle arrives at a station it usually stops. Such rules and patterns should be confirmed by control data. However, if no reference data is available, expert comments, logical rules and standard specifications are also part of this process. Based on the size of the bounding box of the trajectories, the types of features (i.e., polygon, polyline, point) can be inferred. Using speed and pattern of movement, it is possible to identify junctions, bus lanes, pedestrian-only routes, one-way roads, no U-turns layouts, and so on. 

The rules and patterns can be recognised using relevant parameters including the speed of the movement (km/h), number of similar/matching segments in a 20 m^2^ area, density of vertices (i.e., number of vertices that belong to one trajectory in a 20 m^2^ area), density of vertices belong to all matching trajectories in a 20 m^2^ area, date, weather condition (classified as only rainy, cloudy and sunny), number of split points of one trajectory, type of the contributor (classified into categories of active, familiar, elementary (new), and beginner (New)). 

The geometry type refers to the OGC standard for spatial data types, which need to be identified. For linear features, predictably, it is much easier to identify the spatial data type. However, polygon features can be possibly considered as a point feature. This is mainly due to the positioning inaccuracy, where for a fixed and small feature an area can be traversed, see Figure 6. The geometrical similarity indicates how close the shape is to its real world equivalent (or reference map equivalent) based on the orientation and the size of the identified feature. Topological relationships, such as inclusion and intersection, refer to the relationships between the identified objects and the other map features which remain invariant irrespective to some transformations, such as rotation or zooming in/out. 

The important parameters in feature extraction are identified using the Random Forest method [59]. The most important parameter is the density of vertices of one trajectory (0.7835% correlation), and the speed of the movement. Understandably the speed of the movement can indicate the travel mode; higher speed can potentially indicate a road feature (with a polyline geometry type) and low speed can refer to a pedestrian or a bicycle rider. The density of split points and the number of vertices in a relatively small area (20 m^2^) can indicate if the trajectory stops or goes around a specific location, which can be an indication of the point or polygon features (such as shopping centre or residential buildings), where the users stay for a while or moves from one spot to other close spots. 

**Figure 9:**
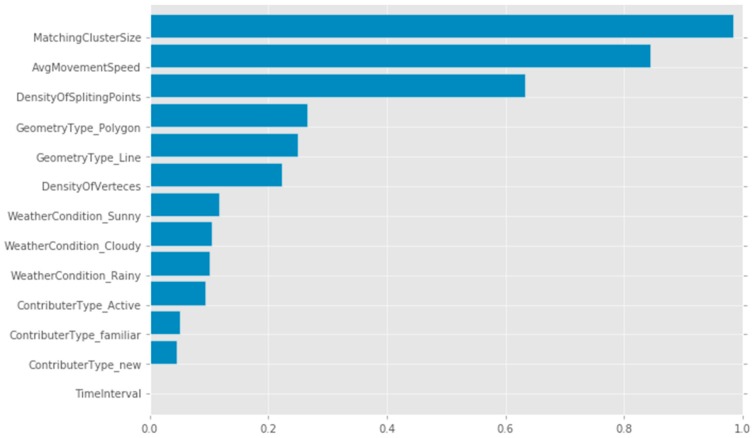
Normalised importance of trajectories’ features for tag identification in Random Forest method.

The least important parameters are the time interval for capturing to sequential points, and the cloudy weather (0.0001, and 0.0003% correlation respectively). The relatively low importance of time interval parameters could be mainly due to the fact that the contributors usually do not change the default settings of their trajectory data capture application software. One of these settings is a 2 s interval for the sequentially captured coordinates. The rainy and sunny weather may have an impact of the travel mode, the speed of the movement and even the shape of trajectory. However, the cloudy weather did not seem to have a similar impact. The normalised importance values are shown in Figure 9.

As it is explained earlier the input dataset is randomly divided into two sets of training and test datasets. This paper applies several supervised machine-learning techniques for classification tasks, including K-Nearest Neighbour (KNN), Logistic Regression, Ridge Classifier, and Random Forest [60]. There are two classification tasks; one is the classification of the geometry type while the other is the tag identification or the classification of geography types. 

**Figure 10:**
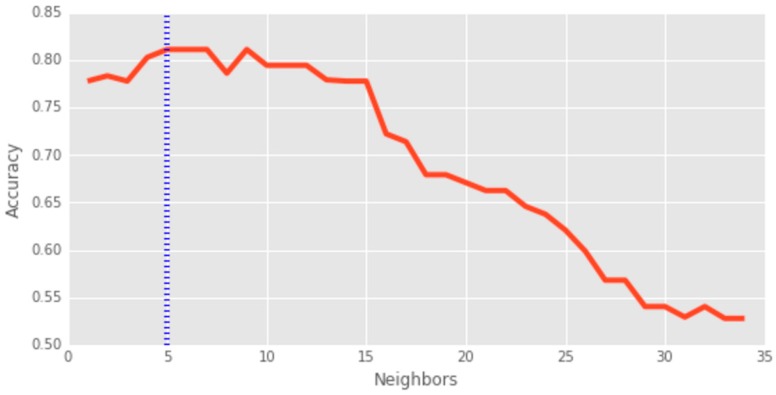
The accuracy of K-Nearest Neighbour (KNN) method to classify the geometry type of trajectories.

For geometry type classification, the best accuracy (81.11%) is achieved by KNN by taking the 5 nearest trajectories (*N* = 5) as shown in Figure 10. For geography types (attributes of the features) the random forest method provides the best accuracy of 87.22% with 20 trees in the ensemble. Despite the high level of accuracy for type classification, the shape, orientation, and the size of the features highly depends on the number of contributed trajectories assigned to that feature. For polyline features, usually representing roads, the shape and orientation can be the same as the cluster of trajectories. For polygon features, the bounding rectangle of the trajectories including a buffering for the walls, represent pathways and emergency exit corridors. However, this does not always generate the actual buildings or physical areas in the real world. This is mainly due to the fact that contributors may not traverse all of the around a building to create a building footprint. Therefore, the spatial area identified by this approach, such as the polygon feature, may cover only some parts of the actual area. This results in only 56.23% accuracy for the results of our approach. 

Using the statistical values, and the normalised importance of the trajectories’ feature, it is possible to understand some patterns and rules. However, such interpretations are only useful for us to make the rules easier to understand. Some of the examples of such rules and patterns which explain the correlation between important factors are listed below. The fixed values, such as 50 km/h, 6 split points or 10 trajectories, are based on the 68% confidence interval, extracted from the training samples.

- If the average speed of the movement is more than 50 km/h and the number of splitting points is less than 6 then the feature is polyline with the tag or attribute, ‘road’.
- If the average speed of the movement is more than 15 km/h, the number of splitting points is less than 10 and the cluster of trajectories include less than 10 trajectories gathering in the area smaller than 500 m^2^ on a nightly basis then the geometry type is polygon and the tag or attribute represents a residential building.

The proposed approach can successfully identify the common tags including the type of the roads (i.e., motorway, bicycle lanes, dual and single carriage roads, one and two-way streets), junctions and residential buildings. However, the proposed approach predictably fails to identify one of the most common tags, i.e., feature name, as the correlation between feature names and the type of the features is almost zero. This shows that the best application of the proposed methods can be in the areas where the geometries of the objects are already inserted by mappers but there is a lack of attributes actually describing the features. This can happen when OSM mappers who are not physically active in a given area or region trace out building outlines from available aerial imagery. Interestingly, it seems that there is no significant impact for being a familiar or expert contributor on the achievable accuracies of the results. 

## 5. Conclusions and Future Work

Despite the widespread use of OpenStreetMap (OSM) data, its completeness and reliability are still under question as the percentage of active and expert registered contributors is still relatively low. This paper has proposed an automated methodology for OSM data contribution based on the processing of raw movement trajectories of contributors and volunteers to OSM. These raw movement trajectories are uploaded to OSM by contributors. This methodology can minimise data input errors and also improve the completeness of OSM data in terms of attributes and annotations. Using trajectory data mining techniques, the proposed methodology can identify the geometry and properties of geographical features: where OSM volunteers gathered, where they visited, where they drove, or simply stayed. There are some rules and patterns in clusters of trajectories, which can highlight some of the properties of features, such as the type of the buildings, the road type or classification and the geometry and overall shape of the features. In order to recognise such patterns and rules an inference engine was developed as an ArcGIS add-in which can store, visualise and analyse trajectories and then infer rules and patterns using spatial data-mining techniques. The implementation of the proposed methodology shows that the significant success of this work is in tag identification rather than geometry recognition. This could be a very helpful contribution to OSM as this could be of great assistance in cases where objects are inserted to the OSM database but there are not enough tags supplied to describe the feature sufficiently. This approach could also minimise the human errors made by new contributors in OSM and low-quality data inserts. Our results indicate that the achievable accuracy of the proposed methodology is not significantly affected by the type or experience of the contributors.
